# The study of laccase immobilization optimization and stability improvement on CTAB-KOH modified biochar

**DOI:** 10.1186/s12896-021-00709-3

**Published:** 2021-08-05

**Authors:** Zhaobo Wang, Dajun Ren, Shan Jiang, Hongyan Yu, Yaohui Cheng, Shuqin Zhang, Xiaoqing Zhang, Wangsheng Chen

**Affiliations:** 1 College of Resource and Environmental Engineering, Wuhan University of Science and Technology, Wuhan, 430081 China; 2 Hubei Key Laboratory for Efficient Utilization and Agglomeration of metallurgic Mineral Resources, Wuhan University of Science and Technology, Wuhan, 430081 Hubei China

**Keywords:** Laccase, Modified biochar, Immobilization optimization, Stability improvement

## Abstract

**Background:**

Although laccase has a good catalytic oxidation ability, free laccase shows a poor stability. Enzyme immobilization is a common method to improve enzyme stability and endow the enzyme with reusability. Adsorption is the simplest and common method. Modified biochar has attracted great attention due to its excellent performance.

**Results:**

In this paper, cetyltrimethylammonium bromide (CTAB)-KOH modified biochar (CKMB) was used to immobilize laccase by adsorption method (laccase@CKMB). Based on the results of the single-factor experiments, the optimal loading conditions of laccase@CKMB were studied with the assistance of Design-Expert 12 and response surface methods. The predicted optimal experimental conditions were laccase dosage 1.78 mg/mL, pH 3.1 and 312 K. Under these conditions, the activity recovery of laccase@CKMB was the highest, reaching 61.78%. Then, the CKMB and laccase@CKMB were characterized by TGA, FT-IR, XRD, BET and SEM, and the results showed that laccase could be well immobilized on CKMB, the maximum enzyme loading could reach 57.5 mg/g. Compared to free laccase, the storage and pH stability of laccase@CKMB was improved greatly. The laccase@CKMB retained about 40% of relative activity (4 °C, 30 days) and more than 50% of relative activity at pH 2.0–6.0. In addition, the laccase@CKMB indicated the reusability up to 6 reaction cycles while retaining 45.1% of relative activity. Moreover, the thermal deactivation kinetic studies of laccase@CKMB showed a lower k value (0.00275 min^− 1^) and higher t_1/2_ values (252.0 min) than the k value (0.00573 min^− 1^) and t_1/2_ values (121.0 min) of free laccase.

**Conclusions:**

We explored scientific and reasonable immobilization conditions of laccase@CKMB, and the laccase@CKMB possessed relatively better stabilities, which gave the immobilization of laccase on this cheap and easily available carrier material the possibility of industrial applications.

## Background

Laccase is a widely distributed copper oxidase, which belongs to ligninase like lignin peroxidase (LiP) and manganese peroxidase (MnP) [1]. The active center of laccase is three types of four copper atoms, including one type I copper atom (T1), one type II copper atom (T2) and two type III copper atoms (T3) [2]. The catalytic mechanism of laccase can be roughly described as T1 takes electrons from the oxidized substrate and transfers them to T2/T3; T2/T3 combines with oxygen atom to reduce O_2_ to H_2_O [3]. The substrate spectrum of laccase is very rich, including arylamines, aromatic thiols and substituted phenols, which shows the application potential of laccase in the environmental field [4]. In general, laccase is capable of oxidizing phenolic pollutants, PAHs and contaminating pharmaceuticals, with H_2_O as the only byproduct [5–7]. Although laccase has a good catalytic oxidation ability, free laccase shows extremely high sensitivity to environmental conditions, which means that the stability of laccase is poor under natural conditions. Enzyme immobilization is a common method to improve enzyme stability and endow the enzyme with reusability.

Laccase immobilization means coupling the enzyme to an insoluble carrier matrix, which are traditionally classified into entrapment/encapsulation, covalent bonding, cross-linking and adsorption methods. Among the existing laccase immobilization methods, adsorption is the simplest and common method. In recent years, bentonite, activated carbon, metal organic frameworks (MOFs), biochar and chitosan are some attractive carrier materials [8–13].. Biochar has attracted great attention due to its large specific surface area, porous, low-cost and easy availability. In additon, another attractive reason for biochar is that it can can be modified by certain methods to enhance its performance, such as alkali modification and acid modification. Jin et al. [14] used KOH to modify the biochar, and the results showed that the specific surface area increased from 14.4 m^2^/g to 49.1 m^2^/g. The adsorption capacity of modified biochar has increased by almost 1.5 times compared with before, and the maximum adsorption capacity has been increased from 21.12 mg/g to 50.71 mg/g. Peng et al. [15] used phosphoric acid to modify biochar, and the results showed that the specific surface area of the modified biochar was larger, and the adsorption performance of Cu^2+^ and Cd^2+^ was better.

In recent years, there have been many examples of improving the stability and reusability of laccase by adsorption-immobilized laccase, such as Taheran et al. [16] immobilized laccase onto homemade polyacrylonitrile-biochar composite nanofibrous membrane, and the results showed a good storage, temperature and pH stability improvement. In addition, the immobilized laccase retained 50% of relative activity after 7 ABTS oxidation cycles. Moreover, Li et al. [17] achieved a good immobilization of laccase on maple biochar via adsorption method. The results showed the enzyme loading was 11.14 mg/g and the thermostability of laccase was significantly improved. The maple biochar immobilized laccase retained 30% of relative activity after 7 reaction cycle. However, the studies on the improvement of stability and reusability of immobilized laccase on cetyltrimethylammonium bromide (CTAB)-KOH modified biochar (CKMB) have not been reported. In this work, the laccase was immobilized onto CKMB via simple adsorption method, the optimization of immobilization was studied via Design-Expert 12 and the properties of free laccase (FL) and immobilized laccase (laccase@CKMB) were compared. In addition, the reusability performance of laccase@CKMB was investigated.

## Results

### Characterization analysis

**Fig. 1:**
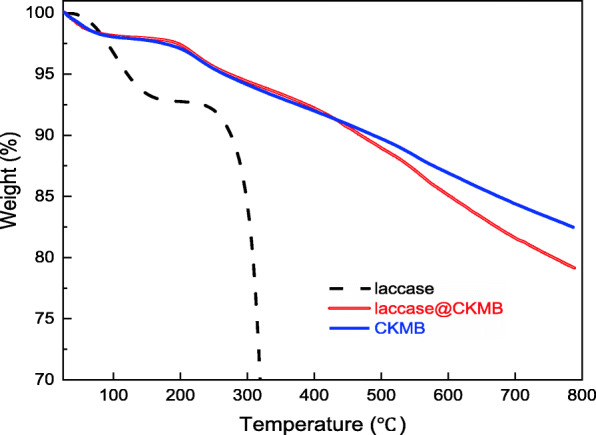
The TGA curve of CKMB and laccase@CKMB

The thermogravimetric analysis (TGA) curves are performed to examine the thermal properties of free laccase (FL), CKMB and laccase@CKMB with a constant heating rate of 10 °C/min from 25 to 800 °C under N_2_ (Fig. 1). There are two weight-losses from 30 to 150 °C and > 250 °C for the FL, the first weight-loss is corresponding to the removal of structural water and the second weight-loss is corresponding to the pyrolysis of laccase. The curves of CKMB and laccase@CKMB are similar and both have good thermal stability. The weight-losses of CKMB and laccase@CKMB can be also divided into 30 to 150 °C and > 250 °C. The first stage is mainly the loss of crystallization or free water in the structure, the second stage is mainly the pyrolysis of CKMB and laccase, this is also the reason why the weight-loss of laccase@CKMB is greater. In addition, the change of temperature range may be due to the high thermal stability of laccase@CKMB, which may be related to the thermal decomposition of laccase and the effective immobilization of laccase [18].

**Fig. 2:**
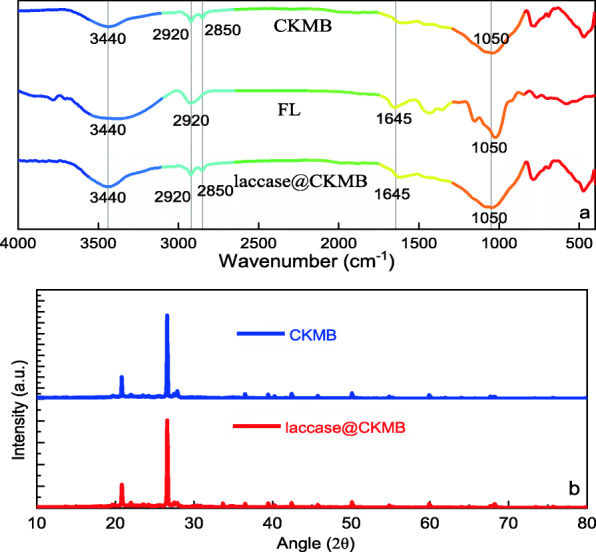
(**a**) The FTIR spectra of FL, CKMB and laccase@CKMB. (**b**) XRD patterns of CKMB and laccase@CKMB

According to the analysis of the infrared spectrum (Fig. 2a), the common peak positions of CKMB, FL and laccase@CKMB are at 3440, 2920 and 1050 cm^− 1^. Among them, the broad peak at 3440 cm^− 1^ is the stretching vibration peak of the intermolecular hydrogen bond (υ_OH_) of multi-molecule association [19]; the peak at 2920 cm^− 1^ is the stretching vibration peak of υ_asCH_, indicating that it contains a saturated hydrocarbon group -CH2 - [20]; The broad peak at 1050 cm^− 1^ is the stretching vibration peak of the hydroxyl group (υ_C-O_), which may contain primary alcohols. The peak at 1645 cm^− 1^ in the infrared spectrum of FL represents the stretching vibration peak of C-H, indicating that it contains unsaturated hydrocarbon groups C=C [21]; the peak at 2850 cm^− 1^ in the infrared spectrum of CKMB is the stretching of υ_sCH_, indicating that it contains saturated hydrocarbon group -CH2-, which is a sign of the successful grafting of the quaternary ammonium cation in CTAB to the surface of biochar. In addition, the infrared spectra of laccase@CKMB all contained the above peaks, indicating that laccase was successfully immobilized on the surface of biochar. The CKMB and laccase@CKMB are characterized by X-ray diffraction. The XRD patterns (Fig. 2b) of CKMB and laccase@CKMB samples do not show any significant differences, indicates that the immobilization process of laccase did not affect the structure of CKMB. In addition, Biochar has a disordered structure of amorphous phase, which is mainly caused by the uneven pyrolysis of molecules in the pyrolysis process of biochar [22]. In short, the results of XRD and FTIR help to some extent prove that laccase is well loaded on CKMB.

**Fig. 3:**
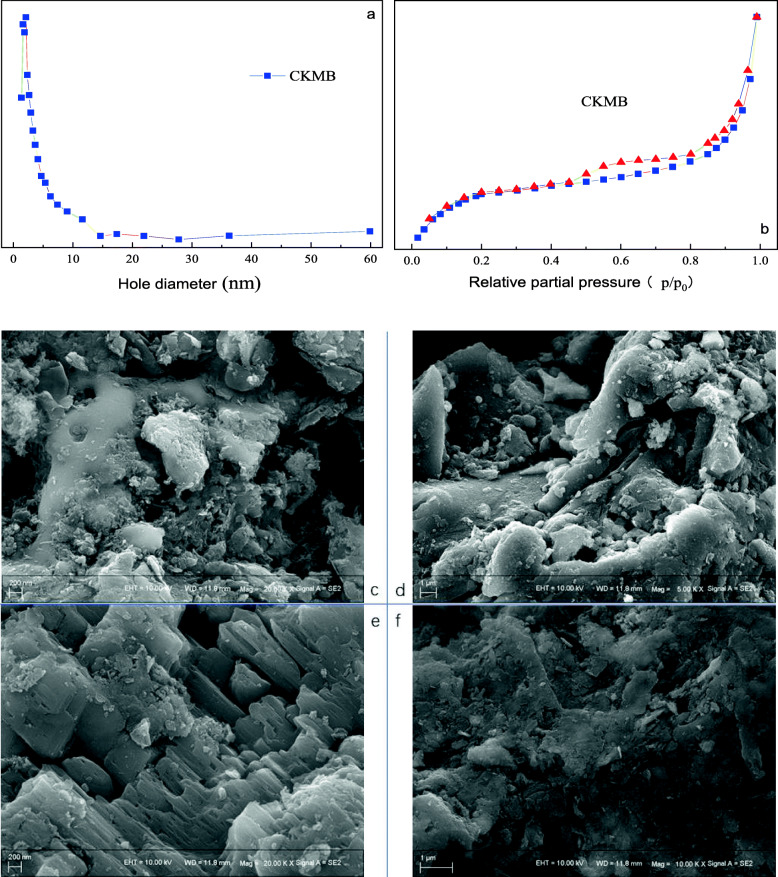
(a) The pore size distribution of CKMB. (b) The N_2_ adsorption-desorption curve of CKMB. Scanning electron micrograph of (c and d) CKMB, (e and f) laccase@CKMB

The results of BET (Fig. 3a) show the S_BET_ of CKMB was 221.352 m^2^/g, S_mic_ was 87.135 m^2^/g, V_total_ was 0.459 m^3^/g, V_mic_ was 0.160 m^3^/g, and the average pore size was 7.64 nm. It can be seen fromFig. 3b that the adsorption-desorption curves of CKMB do not overlap 100%, the adsorption curve has a clear hysteresis, and there is an inflection point in the low phase region. According to the capillary aggregation phenomenon, this indicates that CKMB contains a small amount of mesopores and macropores. The scanning electron micrographs of CKMB as well as laccase loaded CKMB are given as Fig. 3 c,d,e and f. Figure 3 c/d and Fig. 3 e/f represent CKMB before and after laccase immobilization, respectively. The surface texture of the immobilized laccase did not change clearly. However, agglomeration phenomenon was observed after the laccase was immobilized with CKMB, which could be attributed to the change in surface charge [23].

### Optimal immobilization conditions of laccase@CKMB

#### The influence of single-factor on the immobilization effect

**Fig. 4:**
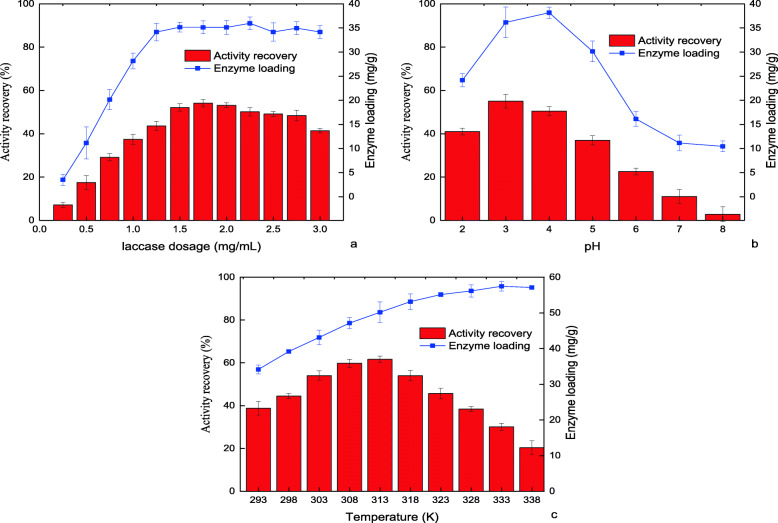
The effect of (a) laccase dosage, (b) pH and (c) temperature on the immobilization effect

The laccase dosage is the parameter directly related to the cost, and a relatively suitable laccase dosage means a reduction in cost. It could be seen from Fig. 4a that the AR and enzyme loading of laccase@CKMB had the similar changing trends. When the laccase dosage was increased from 0.25 mg/mL to 1.25 mg/mL, the enzyme loading was increased from 3.5 mg/g to 34.2 mg/g. At this time, the enzyme loading was close to the peak value. As shown in Fig. 4b, in the pH range of 2.0–8.0, the AR of laccase@CKMB reached a maximum of 55.1% near pH 3.0. At pH 4.0, the enzyme loading reached a maximum of 38.2 mg/g, and then showed a downward trend, which was very obvious. The most influential factor in actual industrial applications is the reaction environment temperature. As shown in Fig. 4c, the AR changes of laccase@CKMB all presented a “bell-shaped” distribution. The AR of laccase@CKMB reached a maximum of 61.7% at 313 K. The enzyme loading was increasing with temperature, rising to the maximum value of 57.5 mg/g at 333 K.

#### Optimization of laccase immobilization via design-expert

On the basis of above single-factor experiment results, we designed a three-factor three-level response surface test of laccase dosage (A, 1–3 mg/mL), pH (B, 2.0–4.0) and temperature (C, 303–323 K) using Design-Expert 12 (Stat-Ease, Inc., Minneapolis, MN, USA). Table 1 listed the coded (− 1, 0, + 1) and actual values of the independent factors, and a total of 17 experimental runs were shown in Table 2, including 5 center points per block.

**Table 1:** The coded levels of the independent variables in the application of the Box-Behnken design for laccase@CKMB

Factor	Name	Units	Type	Minimum	Maximum	Coded Low	Coded High	Mean	Std. Dev.
A	laccase dosage	mg/mL	Numeric	1.0	2.5	−1	+ 1	1.75	0.7071
B	pH		Numeric	2.0	4.0	−1	+ 1	3.0	0.7071
C	temperature	K	Numeric	303	323	−1	+ 1	313	7.07

**Table 2:** Box-Behnken experimental design and recovery activity of laccase@CKMB

Run	A	B	C	Y	
	laccase dosage	pH	temperature	activity recovery (%)	
1	1	−1	0	41.23	
2	0	1	1	40.65	
3	0	0	0	61.73	
4	0	1	−1	39.23	
5	−1	0	1	32.84	
6	1	1	0	46.29	
7	0	0	0	63.02	
8	0	0	0	60.29	
9	1	0	1	36.67	
10	−1	0	-1	43.49	
11	0	-1	-1	40.29	
12	0	0	0	62.52	
13	0	0	0	59.99	
14	-1	1	0	44.24	
15	-1	-1	0	40.23	
16	1	0	-1	42.07	
17	0	-1	1	30.61	

We adopted the central combination model to conduct a three-factor three-level response surface analysis test. The results from Table 3 showed that factor coding is coded and sum of squares is type III–Partial. In addition, the Model F-value of 98.88 implied the model is significant. There was only a 0.01% chance that an F-value this large could occur due to noise. *P*-values < 0.0500 indicate model terms are significant. In this case B, C, BC, A^2^, B^2^, C^2^ are significant model terms (P-values > 0.1000 indicated the model terms were not significant). Where A was laccase dosage, B was pH and C was temperature. The Lack of Fit F-value of 1.42 and P-value of 0.3595 indicated the Lack of Fit is not significant relative to the pure error, which proved the mathematical regression model was reliable. It could also be seen from the F-value that the order of the influence of each factor on the change of the AR in the experiment was: temperature (C) > pH (B) > laccase dosage (A). The quadratic polynomial regression model was estimated using Design-Expert 12 for the AR of laccase@CKMB is Y = 61.51 + 0.6825A + 2.26B − 3.04C + 0.2625AB − 1.31AC + 2.78BC − 8.72A^2^ − 9.79B^2^ − 14.02*C*^2^.

**Table 3:** The analysis of variance (ANOVA) for the fitted quadratic polynomial model of laccase@CKMB

Source	Sum of Squares	df	Mean Square	F-value	p-value	
Model	1879.61	9	208.85	98.88	< 0.0001	significant
A	3.73	1	3.73	1.76	0.2258	
B	40.73	1	40.73	19.28	0.0032	
C	73.87	1	73.87	34.97	0.0006	
AB	0.2756	1	0.2756	0.1305	0.7286	
AC	6.89	1	6.89	3.26	0.1138	
BC	30.80	1	30.80	14.58	0.0066	
A^2^	320.16	1	320.16	151.58	< 0.0001	
B^2^	403.76	1	403.76	191.16	< 0.0001	
C^2^	827.92	1	827.92	391.98	< 0.0001	
Residual	14.79	7	2.11			
Lack of Fit	7.64	3	2.55	1.42	0.3595	not significant
Pure Error	7.15	4	1.79			
Cor Total	1894.39	16				

**Fig. 5:**
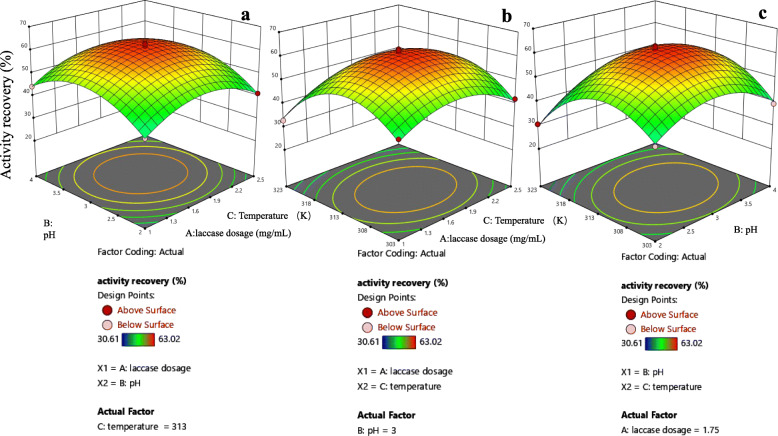
(a) The effect of laccase dosage (A) and pH (B) on the immobilization effect. (b) The effect of laccase dosage (A) and temperature (C) on the immobilization effect. (c) The effect of pH (B) and temperature (C) on the immobilization effect

In order to describe the individual and cumulative effects of independent variables on the response, Design-Expert 12 was used to graphically represent the fitted polynomial equations as response surfaces and contour plots (Fig. 5). Based on the RSM and estimated regression coefficient, we gave the optimal value of the selected variable. The AR of laccase@CKMB was related to laccase dosage to a certain degree (Fig. 5a and b). Excessive laccase dosage could lead to lack of space between molecules (steric hindrance), which resulted in mass transfer limitation [24]. This obstacle was due to too many enzymes on the surface of the carrier, which limited the dispersion of substrates and products [25]. The effect of pH on the AR of laccase@CKMB was shown in Fig. 5a and c. It could be seen that pH had a great influence on the response surface. It is well known that the catalyst will be dissociated into an acid-catalyzed state or a base-catalyzed state after bonding with the substrate, and only a few catalysts can have both two dissociation states [26]. Laccase belongs to this kind of catalyst with two dissociation states, that is, the active group of laccase can be dissociated into two different states of proton donor or proton acceptor, respectively [27]. The measured pH_pzc_ of CKMB is 5.5–6.0, and the isoelectric point of laccase is approximately pH 3.0. Therefore, when the pH is between 2.0–3.0, the surfaces of laccase and CKMB are both positively charged, causing electrostatic repulsion. In addition, there is electrostatic attraction between the laccase and the carrier at pH range of 3.0–4.0. Therefore, the electrostatic attraction is strongest at pH 4.0, and the enzyme loading reaches its maximum. However, the AR results of laccase@CKMB indicate that the dissociation state of the enzyme molecule at pH 3.0 fits best with the carrier, so that CKMB can retain laccase activity to the greatest extent [28]. The temperature had significant influence in the response (Fig. 5b and c), which meaned the AR of laccase@CKMB was very dependent on the temperature. Generally, in order to accelerate the enzymatic reaction or make the enzymatic reaction proceed smoothly, a certain temperature is often increased to provide sufficient energy (reaction activation energy) for the enzymatic reaction. However, under different temperature conditions, the activity of enzyme molecules may change significantly, which affects the catalytic reaction. If the temperature is too high (in this article, when the temperature exceeds 313 K), the three-dimensional conformation of the enzyme may change, which means that some unstable groups may be oxidized with sufficient energy. In addition, too low temperature could also cause the enzyme activity to decrease or even dormancy, but it was not completely inactivated like at high temperature, because the structure of the enzyme has not changed. In addition, the predicted optimal experimental condition evaluation suggestion via Design-Expert 12 was about laccase dosage 1.78 mg/mL, pH 3.1 and 312 K. Under these conditions, the activity recovery of laccase@CKMB was the highest, reaching 61.78% .

### Stability of FL and laccase@CKMB

#### Storage, pH stability and reusability analysis

**Fig. 6:**
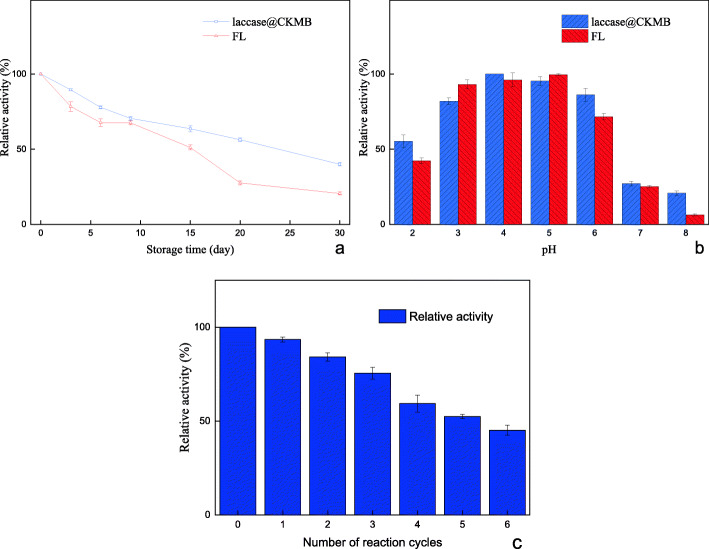
(a) Storage stability of free laccase and laccase@CKMB. (b) The pH sability of FL and laccase@CKMB. (c) Reusability of laccase@CKMB

As we all know, the storage stability of enzyme is an important factor to consider when developing robust biocatalysts. The storage stability was assessed by storing at 4 °C for 30 days, the RA of laccase@CKMB and FL was determined per few days. It was observed that the RA of FL was 20.19%, and the RA of laccase@CKMB was 39.96% after 30 days as shown in Fig. 6a. The result indicated that the laccase@CKMB had better storage stability than FL. As shown in Fig. 6b, the trend of RA of FL and laccase@CKMB did not show significant difference within pH range of 2.0–8.0. FL reached the maximum activity at pH 5.0, but laccase@CKMB reached the maximum relative activity at pH 4.0. In actual production, although FL has excellent catalytic degradation ability, and the degradation products are clean and pollution-free. However, FL cannot be recycled and reused, resulting in high costs. Immobilization is one of the important solutions to this problem, and reusability becomes an important evaluation index. The reusability of laccase@CKMB during 6 reaction cycles was investigated via ABTS as substrate. The results showed a almost half of the activity losss after 6 cycles and retained about 45.1% of RA (Fig. 6c).

#### Thermostability and thermal deactivation kinetics model

**Fig. 7:**
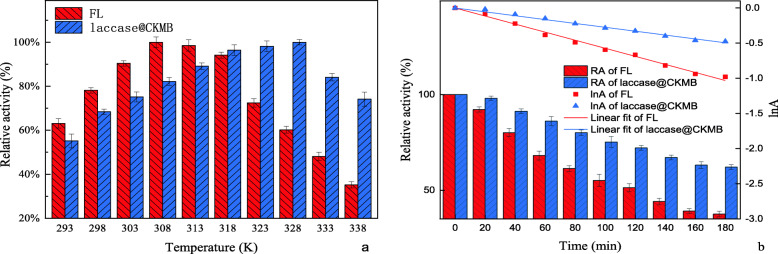
(a) Thermostability of FL and laccase@CKMB. (b) Thermal deactivation kinetics fitting curve and thermal tolerance of FL and laccase@CKMB (333 K)

As shown in Fig. 7a, we determined the RA change at 293–338 K, it was observed that the RA of laccase@CKMB was greater than 50%, and the maximum RA was obtained at 328 K; The minimum RA of FL was about 35%, and the maximum RA was obtained at 308–313 K. The Thermal deactivation kinetics fitting curve and thermal tolerance of FL and laccase@CKMB at 333 K was shown in Fig. 7b. The thermal tolerance of the laccase@CKMB was significantly better than that of FL. The RA of FL and laccase@CKMB both showed a significant downward trend with time. However, the rate of decrease in RA of FL was significantly greater than that of MBL. The RA of FL (33.6%) and laccase@CKMB (60.1%) reached a minimum at 180 min.

Table 4 depicted the comparison of deactivation rate constants (k), half-life (t_1/2_), and the R value of FL and laccase@CKMB at 333 K. The k value of laccase@CKMB (0.00275) was much lower when compared to FL (0.00573), which also conferred 2 folds enhanced half-life than that of FL. In addition, the experimental data obtained for both FL and laccase@CKMB were adequately represented via the first-order model in Fig. 7b, with an excellent correlation for both curves (R^2^ = 0.998 and 0.996 for FL and laccase@CKMB, respectively).

**Table 4:** Thermal deactivation kinetics model parameters for FL and laccase@CKMB at 333 K

Method	FL	laccase@CKMB
k (min^−1^)	0.00573	0.00275
t_1/2_(min)	121.0	252.0
R	0.998	0.996

### The comparison of enzymatic parameters of different immobilized laccases

The results of enzyme loading, activity recovery and stability of the immobilized laccase are compared with the results of similar research previously reported in the literature of recent years (Table 5). In recent years, there are few data about the enzyme loading and activity recovery of immobilized laccase reported in the relevant literature, but it also can be seen from Table 5 that the enzyme loading (57.5 mg/g) and activity recovery (61.78%) of this study is comparable to the data reported in other studies. For the comparison of stability, we pioneered the design of a stability comparison method, which is to compare stability parameters greater than 50% in the relevant literature. It can be inferred from Table 5 that laccase@CKMB has a good storage stability and thermal stability, and possesses a wide pH range and multiple reuse times. It is worth noting that the carrier used in this study is prepared from agricultural solid waste-rice straw, and the CKMB has the advantages of low cost and easy preparation. The above conclusions all prove the potential of laccase@CKMB in industrial applications (such as wastewater treatment, dye bleaching, etc.).

**Table 5:** The comparison of enzymatic parameters of different immobilized laccases

Carrier		polyacrylonitrile-biochar	pinewood nanobiochar	Fe_3_O_4_@ZIF-8	calcium/copper alginate beads	E-CLEA	PVDF/MWCNT membrane	polyacrylamide-alginate cryogel	CTAB-KOH modified biochar
Year		2017	2018	2019	2019	2019	2021	2021	2021
Source of Laccase		*Trametes versicolor*	*Trametes versicolor*	*B. amyloliquefaciens LC02*	*Cyberlindnera fabianii*	*Trametes versicolor*	*Trametes hirsuta*	*Trametes versicolor*	*Trametes versicolor*
Enzyme loading (mg/g)		10.1	–	–	–	–	30.4	68.7	57.5
Activity recovery (%)		–	–	75.5	75	–	38.31	–	61.78
Stability (RA > 50%)	Storage	> 30 days	25 days	> 10 days	> 21 days	> 20 days	–	–	> 20 days
	pH	3.0–8.0	3.0–5.0	–	3.0–9.0	4.0–5.0	–	2.5–4.0	2.0–6.0
	Thermal	20–60 °C	20–60 °C	60–80 °C	30–70 °C	25–55 °C	20–70 °C	30–70 °C	30–66 °C
	Reuse	6 cycles	3 cycles	5 cycles	3 cycle	20 cycles	2 cycles	7 cycles	5 cycles
Reference		[29]	[30]	[31]	[32]	[33]	[34]	[35]	This work

## Discussion

When laccase dosage was 1.75 mg/mL, the AR reached a maximum of 54.1%, and then the AR showed a downward trend. This phenomenon indicated that the carrier was saturated due to excess enzyme in the solution. Similar observations had been made in previous study [36], they observed a decrease in the activity recovery of bentonite-derived mesoporous materials immobilized laccase when the laccase dosage exceeded 2 mg/mL. The overload of FL on the surface of the carrier will cause the congestion or crowding of enzyme molecules [37]. The measured pH_pzc_ of CKMB is 5.5–6.0, and the isoelectric point of laccase is approximately pH 3.0. Therefore, when the pH was between 2.0–3.0, the surfaces of FL and CKMB are both positively charged, which lead to the electrostatic repulsion between the laccase and the carrier; When the pH was between 3.0–6.0, there was electrostatic attraction between the laccase and the carrier. As the pH increased, the negative charge of the laccase gradually increased, and the positive charge on the surface of the carrier gradually decreased. Therefore, when the pH is 4.0, the electrostatic attraction is the strongest, and the enzyme loading reaches the maximum (38.2); When the pH was greater than 6.0, the surfaces of the FL and CKMB were negatively charged, and there was electrostatic repulsion between laccase and carrier, so the adsorption capacity was reduced. There is no synchronization relationship between AR and enzyme loading, which was mainly because the load process of FL on CKMB was an endothermic reaction process. The increase in temperature provided more favorable conditions for the loading of laccase [38]. However, the increase in temperature lead to a rearrangement of the three-dimensional conformation of laccase, which meaned a decrease in enzyme activity.

The optimal pH of laccase@CKMB changed from 5.0 (FL) to 4.0, indicating that an electrostatic interaction occurred between the protein and the matrix microenvironment. In addition, changes in the dissociation and ionization state of the enzyme during the immobilization process may have caused changes in pH. Moreover, the decrease in the RA of both FL and laccase@CKMB at high pH (more than 7.0) could be attributed to the inhibition of the enzyme, which is caused by the bonding of the hydroxide ions to Cu of the active site of enzyme. On the other hand, the inappropriate pH of the solution might cause the amino acid originally inside the laccase to be exposed to the environment, which leads to a decrease in laccase activity. This indicated that laccase was more suitable for the pH range of 2.0–6.0, but laccase @CKMB showed a higher tolerance under alkaline conditions. The reason for the general reusability may be that we used a milder physical adsorption method to immobilize laccase. Therefore, the binding force between FL and the active site of CKMB is not strong enough. After multiple reaction cycles and washing, a large amount of FL falled off the surface of CKMB, resulting in a decrease in enzyme activity. The similar decrease was reported by Imam et al., their rice straw biochar immobilized laccase demonstrated operational stability up to 6 cycles while retaining 40% of the RA [39]. However, due to the cheap and easy availability of CKMB and the gentle and simple immobilization method, the performance of laccase@CKMB was attractive enough, which also gave laccase@CKMB the potential for industrial applications.

As for thermostability, the RA range of FL changed significantly more than laccase@CKMB, this is because the laccase@CKMB can improve the stability of protein tertiary structure to a certain extent, thereby restricting the mobility of enzyme protein molecules in the system, so that laccase can still maintain high activity when the external environment temperature changes [40]. The results showed that the laccase@CKMB can protect the structural stability of the enzyme molecule. At high temperatures, the three-dimensional conformation of the enzyme protein was prone to multi-directional irregular stretching, which will expose some of its reactive groups and even active sites. This may lead to the polymerization of the protein, or the change of the spatial folding order, resulting in the inactivation of laccase. The immobilization of FL on CKMB reduces the fluidity of laccase to a certain extent, and indirectly improved the stability of laccase molecular conformation. At the same time, in the pore space of CKMB, the independent space where FL was located relatively lags behind the heat reaction process, which also played a certain protective effect on laccase protein and enhances its thermostability.

## Conclusion

This paper explored the adsorption-immobilization of laccase using the CTAB-KOH modified rice straw biochar as a carrier. The surface microscopic characteristics and chemical group characteristics of CKMB and laccase@CKMB were studied by various characterization methods. In addition, we gave the optimal immobilization conditions for laccase@CKMB (the predicted optimal immobilization conditions via Design-Expert 12 were laccase dosage 1.78 mg/mL, pH 3.1 and 312 K), which had a good stability improvement. This provided the possibility of industrial application for the immobilization of laccase through this cheap and easily available carrier material.

## Methods

### Chemicals

*Trametes versicolor* laccase (0.99 U/mg) and (3-ethylbenzothiazoline-6-sulfonate) diammonium salt (ABTS, ≥ 98%) were purchased from from Sigma-Aldrich. Bovine serum albumin (BSA), coomassie brilliant blue G-250, KOH, cetyltrimethylammonium bromide (CTAB), phosphoric acid, sodium dihydrogen phosphate, disodium hydrogen phosphate were purchased from Sinopharm Group Chemical Reagent Co., Ltd. (analytical grade). The rice straw comes from Lianyungang, Jiangsu Province.

### Characterization

Perform thermogravimetric analysis (TGA) on a TGA55 thermal analyzer, and heat the sample in a continuous-flow of nitrogen (flow rate is 80 mL/min) at a rate of 10 °C/min from 25 to 800 °C. Fourier transform infrared spectroscopy (FT-IR) was performed on a Thermo Scientific Nicolet 6700 Fourier transform infrared spectrometer, and the scanning range was 4000 cm^− 1^-400 cm^− 1^. X-ray diffraction (XRD) analysis was performed by Bruker D8A X-ray diffractometer using Cu-Kα radiation (tube voltage: 40 KV; current: 40 mA; scan angle 2θ range: 5 to 80°; scan rate: 5 °/min; wavelength: 0.15406 nm). The nitrogen adsorption-desorption isotherm (BET) was measured on the TriStar II 3flex surface area and porosity analyzer. And the Scanning electron microscope (SEM) studies were carried out in a Zeiss Merlin Compact.

### Optimal immobilization conditions of CTAB-KOH modified biochar immobilized laccase

The modification of biochar was carried out by the method recorded in our previously published article [41]. In brief, The pretreated rice straw was heated to 600 °C in a vacuum tube furnace and kept for 4 h to prepare biochar. Then, the KOH activated biochar was placed in CTAB solution, stirred for 24 h and dried at 80 °C for 48 h to prepare CTAB-KOH modified biochar (CKMB). In order to explore the optimal immobilization conditions of laccase immobilized on CKMB, a series of batch single-factor experiments were completed via adsorption method. 200 mg of CKMB was added to 0.25–3 mg/mL laccase solution (pH 2.0–8.0), then the mixture was shaken at 293–338 K for 4 h. At last, the centrifuged precipitate was dried at 313 K for 48 h to determine the enzyme loading and enzyme activity. Then, the response surface methodology (RSM) and 3-factor Box-Behnken design via Design-Expert 12 (Stat-Ease, Inc., Minneapolis, MN 55413, USA) were used to optimize the immobilization conditions of laccase, where laccase dosage (A), pH (B) and temperature (C) were the 3 independent factors selected for the activity recovery of immobilized laccase (Y) as design response.

### Determination of enzyme loading and enzyme activity

**Fig. 8:**
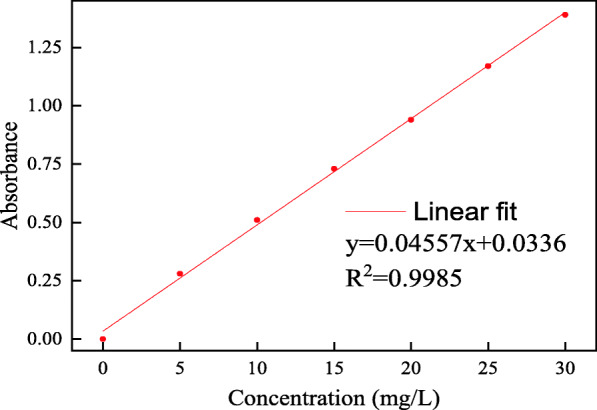
The fitting curve of standard enzyme solution (BSA)

The BSA was used as the standard protein, the protein concentration in the enzymatic extracts was determined by the Bradford method [42]. The absorbance at 595 nm was measured on a UV-2550 UV-Vis Spectrophotometer after 5–20 min. The absorbance showed a good linear relationship with the concentration of standard protein solution, the correlation coefficient R^2^ was 0.9985, and the fitting curve was y = 0.04557x + 0.0366 (Fig. 8). The calculation formula of enzyme loading is as follows:

1 $$ G=\frac{\left({C}_0-{C}_1\right)\bullet V}{M} $$

where G is the enzyme loading (mg/g), C_0_ is the initial protein concentration (mg/mL), C_1_ is the protein concentration in the supernatant (mg/mL), V is the initial protein solution volume (mL), and the M is the mass of the prepared immobilized laccase (g).

The activity of laccase was determined by monitoring the oxidation of ABTS substrate (0.5 mmol / L ABTS). The reaction mixture (1.5 mL of H_3_PO_4_/Na_2_HPO_4_ buffer solution, 1 mL of enzyme solution and 0.5 mL ABTS) was incubated for 3 min in room temperature. The absorbance value of the mixed solution at 420 nm was immediately measured, and the activity was calculated by the Lambert-Beer principle [43]. Generally, an activity unit (U) is defined as the amount of enzyme required to consume 1 μmol of substrate in 1 min [44]. The calculation formula of specific activity (SA, U/mg), activity recovery (AR, %) and relative activity (RA, %) of laccase is as follows:

2 $$ \mathrm{SA}=\frac{A}{m} $$

3 $$ \mathrm{AR}=\frac{SA_i}{SA_f}\times 100\% $$

4 $$ \mathrm{RA}=\frac{A}{A_{max}}\times 100\% $$

Where A (U) is th activity of laccase, and m (mg) is the mass of free laccase (FL) or the enzyme amount of the CTAB-KOH modified biochar immobilized laccase (laccase@CKMB); SA_i_ (U/mg) is the specific activity of laccase@CKMB and SA_f_ (U/mg) is the specific activity of FL; Amax (U) is the maximum activity measured from this set of experiments.

### Stability of FL and laccase@CKMB

The relative activity (RA) of FL and laccase@CKMB was measured at 4 °C per 5 days, and the RA within 30 days reflected the storage stability. The pH stabilities of FL and laccase@CKMB were compared by immersing them in H_3_PO_4_/Na_2_HPO_4_ buffer solution in the pH range 2.0–8.0 for 1 h at room temperature. The reusability of laccase@CKMB was investigated in the catalytic oxidation reaction of ABTS with laccase for 6 reaction cycles. FL and laccase@CKMB were subjected to heat treatment in a water bath at different temperatures (293 K - 338 K). The RA of FL and laccase@CKMB was measured to determine thermostability. In addition, the FL and laccase@CKMB were kept in a 333 K water bath for 3 h (enzyme activity was measured every 20 min) to obtain the thermal deactivation kinetics model, which was given to study their thermal tolerance.

The thermal deactivation of laccase was described as a “one step-two states” process where the active form was transformed in inactive form by a first order unimolecular irreversible reaction [45]. Therefore, the thermal deactivation kinetics model of FL and laccase@CKMB was described by the simple exponential equation of a first-order process. In addition, the half life (t_1/2_) of the enzyme activity was next estimated at 333 K.

The thermal deactivation kinetics equation of first-order model [46]:

5 $$ \ln \frac{A_t}{A_0}=\mathrm{lnA}=-\mathrm{kt} $$

The equation of half-life [47]:

6 $$ {\mathrm{t}}_{1/2}=\frac{\mathit{\ln}2}{k} $$

Where, A_t_ is the enzyme activity at time t, A_0_ is the initial enzyme activity, A is the relative activity at time t, k is deactivation rate constants, t_1/2_ is half-life.

## Data Availability

All data generated and analyzed in this study are included in this published article.

## Abbreviations

CTAB: cetyltrimethylammonium bromide
CKMB: CTAB-KOH modified biochar
laccase@CKMB: CKMB immobilize laccase
FL: free laccase
ABTS: (3-ethylbenzothiazoline-6-sulfonate) diammonium salt
BSA: Bovine serum albumin
TGA: Thermal gravimetric analyses
FT-IR: Fourier Transform Infrared Spectroscopy
XRD: X-ray diffraction
BET: N_2_ adsorption-desorption isotherms
SEM: Scanning electron microscope
AR: activity recovery
RA: relative activity
E-CLEA: entrapped cross-linked enzyme aggregate
PVDF: Polyvinylidene fluoride (PVDF)
MWCNT: modified with multi-walled carbon nanotubes
